# Transition of patients with mucopolysaccharidosis from paediatric to adult care

**DOI:** 10.1016/j.ymgmr.2019.100508

**Published:** 2019-10-21

**Authors:** C. Lampe, B. McNelly, A.K. Gevorkian, C.J. Hendriksz, T.V. Lobzhanidze, J. Pérez-López, K.M. Stepien, N.D. Vashakmadze, M. Del Toro

**Affiliations:** a HELIOS Dr. Horst Schmidt Kliniken, Wiesbaden, Germany; b Salford Royal NHS Foundation Trust, Salford, United Kingdom; c Research Center for Children's Health, Moscow, Russia; d University of Pretoria, Pretoria, South Africa; e Vall d'Hebron University Hospital, Barcelona, Spain

**Keywords:** Mucopolysaccharidosis, Transition, Multidisciplinary team, Metabolic disease, Case studies, ENT, ear nose and throat, ERT, enzyme replacement therapy, GAG, glycosaminoglycan, GP, general practitioner, HCP, healthcare professionals, MDT, multidisciplinary team, MPS, mucopolysaccharidosis, NHS, National Health Service, NICE, National Institute for Health and Clinical Excellence

## Abstract

Mucopolysaccharidoses (MPS) are rare disorders associated with enzyme deficiencies, resulting in glycosaminoglycan (GAG) accumulation in multiple organ systems. As patients increasingly survive to adulthood, the need for a smooth transition into adult care is essential. Using case studies, we outline strategies and highlight the challenges of transition, illustrating practical solutions that may be used to optimise the transition process for patients with MPS disorders.

Seven MPS case studies were provided by four European inherited metabolic disease centres; six of these patients transferred to an adult care setting and the final patient remained under paediatric care. Of the patients who transferred, age at the start of transition ranged between 14 and 18 years (age at transfer ranged from 16 to 19 years).

While there were some shared features of transition strategies, they varied in duration, the healthcare professionals involved and the management of adult patients with MPS. Challenges included complex symptoms, patients' unwillingness to attend appointments with unfamiliar team members and attachment to paediatricians. Challenges were resolved by starting transition at an early age, educating patients and families, and providing regular communication with and reassurance to the patient and family. Sufficient time should be provided to allow patients to understand their responsibilities in the adult care setting while feeling assured of continued support from healthcare professionals. The involvement of a coordinated multidisciplinary team with expertise in MPS is also key. Overall, transition strategies must be comprehensive and individualised to patients' needs.

## 1 Introduction

Transition is the “purposeful, planned movement of adolescents and young adults with chronic physical and medical conditions from paediatric to adult-oriented healthcare systems” [1]. Transfer is the point at which medical responsibility is handed from paediatrics to adult care [2]. Articles examining transition [[2], [3], [4], [5], [6], [7]], have focused on barriers to successful transition and key components that support the process. With an appropriate transition strategy, patients can be expected to remain engaged with the healthcare system, providing the foundation for patients to manage their medical care, when appropriate, with support from healthcare professionals (HCPs). If the patient is not fully engaged, there is the potential for inadequate disease management, leading to poor social and educational outcomes [4,8,9] and economic and emotional burden for the family [10].

A number of barriers can prevent a successful transition from paediatric to adult healthcare systems and influence continued disease management and treatment compliance:

• Complex clinical needs or medical histories [7,[11], [12], [13], [14]];
• Reluctance of parents to reduce involvement in patient care [3,7,11,[14], [15], [16], [17]];
• Anxiety over expectations and levels of care in the adult care system [3,7,11,14,15,[18], [19], [20], [21]];
• Limited or inappropriate preparation for the adult care system [9,11,[22], [23], [24]];
• Familiarity with a paediatric setting and concerns over the lack of established relationship with adult care multidisciplinary teams (MDTs) [3,7,13,15,17,25,26];
• Reluctance of paediatric teams to pass responsibility to adult care MDTs [3,7,11,[13], [14], [15], [16],^20^];
• Lack of coordination of HCPs, between and within paediatric and adult care teams [3,7,9,11,16,19,27,28];
• Access to adult care specialists with specific disease experience [3,7,9,11,[13], [14], [15], [16],^18^,^19^,^21^,^22^,[29], [30], [31], [32]];
• Concerns over patients' disease management capabilities [13,16,17,20,23,33,34].

Transition strategies should be individualised for each patient's medical, social and emotional needs to counter these barriers. Coordinated transition is of particular importance for patients with complex, progressive, rare diseases requiring input from many HCPs.

One such family of rare diseases, the mucopolysaccharidoses (MPS), is a group of metabolic disorders collectively occurring in 1 in 25,000 live births [35,36]. Caused by mutations affecting the activity of lysosomal enzymes, MPS results in glycosaminoglycan (GAG) accumulation in multiple organ systems [35]. Each of the 13 MPS subtypes is associated with different signs and symptoms depending on the deficient enzyme(s), but commonly affected organ systems include musculoskeletal, neurological, cardiac, respiratory, gastrointestinal, auditory and visual systems [35,[37], [38], [39], [40]]. Because of these complex, severe symptoms, patients with MPS have a reduced quality of life and life expectancy [35,37,38,40,41].

With improvements in diagnosis and advances in medical and surgical treatments, patients with MPS are increasingly surviving into adulthood [[42], [43], [44]]. Given the broad range of symptoms in these patients, transition can be a very compound process, which should be reflected in the flexibility of management strategies. A Spanish research group has reported potential barriers to the successful transition of patients with MPS, such as numerous medical requirements, parental involvement, concerns over capabilities of and relationships with the adult care system, reluctance of paediatricians to relinquish responsibilities to adult care teams, and a lack of coordination [7].

This publication describes case studies from four European inherited metabolic disease centres to showcase examples of transition strategies used for patients with MPS. These cases illustrate MPS symptoms that may have a negative impact on transition, highlighting difficulties and identifying practical steps to resolve these challenges.

## 2 Methods

A group of experts in MPS from inherited metabolic disease centres in Germany, Spain, Russia and the UK met on two separate occasions to exchange experiences of transition management strategies for patients with MPS. These centres all have experience of providing care for adults with MPS, but have different strategies in place for managing the transfer of patients from paediatric to adult care services. During the second meeting, it was agreed that the experiences of managing transition in these centres should be communicated to the scientific community involved in MPS management through a series of case studies. This will provide examples of transition strategies that may be implemented in part or in full in other clinical settings.

A literature review on transition strategies was conducted and a series of teleconferences was organised to collect and discuss the required information, and support publication development. Data and cases studies were provided through written templates, telephone interviews and teleconferences. The four European inherited metabolic disease centres were:

• Mark Holland Metabolic Unit, Salford Royal NHS Foundation Trust, Salford, UK;
• Research Center for Children's Health, Moscow, Russia;
• Vall d'Hebron University Hospital, Barcelona, Spain;
• HELIOS Dr. Horst Schmidt Kliniken, Wiesbaden, Germany.

## 3 Results

### 3.1 Transition strategies

The four centres provided details of transition strategies and a total of seven cases (six case studies illustrating transition strategies in patients who were transferred to adult care, and one case in which the patient was retained under paediatric care). Transition strategies varied across the centres, from a transition lasting several years to a shorter period of weeks, and in one centre an adult care clinician manages adult patients in a paediatric setting, whereas the other settings transfer patients to an adult setting. Summaries of transition preparation and implementation and the regulations that guide transition strategies are presented in Table 1. Information on situations when transfer to adult care may be delayed or not undertaken are also included. Fuller details of each centre's strategies are described in the supplementary material, along with details of leaflets and websites about transition and the adult care setting. All centres use the described strategies to manage patients with a range of metabolic disorders, including MPS. At the centre in Germany, no formal transition procedure is in place, whereas at the other three locations, responsibility is officially transferred from a paediatric to an adult care team at the end of transition.

**Table 1:** Transition strategy summaries from four European inherited metabolic disease centres.

Centre	Salford Royal NHS Foundation Trust, Salford, UK	Research Center for Children's Health, Moscow, Russia	Vall d'Hebron University Hospital, Barcelona, Spain	HELIOS Dr. Horst Schmidt Kliniken, Wiesbaden, Germany
Planning and preparation				
Patient age at start of transition (years)	16–17 (the centre is aiming to reduce this to 14–15, and to start transition education at 12)	17	16–17	NA – patients officially remain under paediatric care, but may start visits with the adult care clinician at age 14
Length of transition	1–2 years, depending on comorbidities, such as neurological deterioration, as well as hospital admissions, newly emerging symptoms, and the patient's capacity to manage the disease	<6 months, and patients formally transferred over a 2-week period	1–2 years, depending on active symptoms, planned surgeries and the patient's capacity to manage the disease	NA
Patient understanding of disease and responsibilities	Assessed during a 1:1 appointment with a metabolic nurse, during which transition passport and ‘Ready Steady Go’ documents are introduced	Assessed at routine appointments	Assessed during the initial transition appointment with a metabolic adult care clinician	Assessed at routine appointments
Transition documents/websites (supplementary material)	Transition passport • Explains transition • Records medical details	Website details for paediatric and adult hospitals	Information on the adult care setting	Website details for HELIOS Dr. Horst Schmidt Kliniken and Society for Mucopolysaccharidoses
	Ready Steady Go • Assesses readiness for transfer to adult care			
	Leaflets • Transition plans • Parent/carer • Mental capacity			
Referral to adult care system	Paediatric teams provide adult care services with a list of patients who are at an appropriate age to start transition, and circulate referral letters for the transition clinic	Paediatric teams contact adult care teams when patients are 17	Paediatric teams refer patients to adult care teams at the age of 16	NA

Implementation				
Transition coordinator(s)	Administrative team and nurse	Paediatric team	Paediatric and adult care metabolic clinicians	Paediatrician and adult care clinicians, with administrative support and a nurse
Core transition team members	Adult and paediatric metabolic specialist nurses	Paediatric treating clinician	Paediatric metabolic team	Rare disease paediatrician
	Metabolic consultant	Adult treating clinician	Adult metabolic treating clinician	Rare disease adult care clinician
	Learning disabilities nurse	Infusion nurse	Psychologist	
	Adult metabolic dietician	Psychologist		
	Physiotherapist			
	Paediatric psychologist			
Transition appointments	Held in a transition clinic every 6 months until patient is ready to transfer to the adult care team	Held during routine 6-monthly appointments	Patient attends transition appointments every 4–6 months, until ready to transfer to adult care team	Patients transitioned to adult care clinician working within the same paediatric setting, with appointments every 6–12 months
	Transition clinic held in two locations to allow flexibility for attending patients		Patients sequentially transferred from paediatric to adult care specialists, depending on symptoms	No formal transition clinic
Assessment of readiness for transfer to adult care	Ready Steady Go documents	Disease management and required follow-up under adult care discussed with patient	Transition checklist	Assessed by clinicians
Transfer of medical records from paediatric to adult care teams	Coordinated by administrative team	Prior to transfer, a full medical examination is conducted and details are provided to the adult care team	Managed by paediatric team and adult care nurses	Paediatric and adult care clinicians work in the same paediatric setting
	Details of medical and surgical procedures, medical requirements, assessments and prescriptions are sent from the paediatric team to the adult metabolic team			
			MDT meetings held to evaluate symptom severity throughout transition	
			Records are electronic and so are available to all treating clinicians	
Infusion management during transition	Coordinated by home care nurses	Managed by paediatric or adult care teams, based on patient circumstances	Managed by outpatient clinic or transition nurse if the patient is not receiving infusions at home	Continues under paediatric team management

Other information				
Regulations and guidelines that govern transition	Patients <16 years cannot be managed by adult care services	Patients <18 years must be managed by a paediatric team	Institutional regulations state that patients >16 years are not to attend paediatric emergency rooms, but may be followed up in outpatient clinics until the age of 18 years, or 20 years in special circumstances	Adult care clinicians may treat paediatric patients, but paediatricians are not usually reimbursed for treating adult patients
	Patients >18 years cannot be managed by paediatricians			
		Patients >18 years must be managed by an adult care clinician		
				Adult patients can stay in paediatric wards
	Process is aligned with NICE guidelines on ‘Transition from children's to adults’ services for young people using health or social care services' (NG43) [45]			
		Russian guidelines for MPS are based on international publications [37,40]		
	Mental Capacity Act 2005 adhered to [46]			
Situations during which transfer to adult care could be postponed or not undertaken	Patients without mental capacity to manage their health may be transferred to adult care, while their parents or carers retain responsibility for decision-making [46]	None - all patients are transferred to adult care at the age of 18	Parents may continue to attend appointments if the patient lacks capacity to make decisions regarding their health	NA – care continues in a paediatric setting under the management of an adult care clinician
			Transition timings may be altered for patients in advanced disease stages under palliative care, or if surgery or other procedures are already planned in a paediatric setting	
	Patients under palliative care may remain under paediatric care >18 years of age			
Numbers of patients with MPS transitioned since 2016	Twelve in total: • Five MPS I • Four MPS II • Three MPS IV	Three in total: • One MPS I • One MPS II • One MPS VI	Seven in total: • One MPS I • One MPS II • One MPS IIIA • One MPS IIIB • Two MPS IV • One MPS VII	Twenty-six between the ages of 14 and 18 years: • Five MPS I • Four MPS II • Six MPS III • Five MPS IV • Six MPS VI

MDT, multidisciplinary team; MPS, mucopolysaccharidosis; NA, not applicable; NHS, National Health Service; NICE, National Institute for Health and Care Excellence.

Some features of transition are common to all centres. Metabolic specialists with experience in managing the MPS disorders are key members of each team, with roles as either coordinators or members of a transition clinic. The transition process is explained to patients and their families in advance of the process starting, and further information is provided via websites or leaflets. Patients are able to tour adult facilities and meet the adult care team prior to transferring from paediatric care, and may attend appointments (or parts of appointments) without their parents or carers if preferred. During transition, the paediatric team is responsible for managing patient queries, except at the HELIOS Dr. Horst Schmidt Kliniken in Germany, where these are managed by the adult care clinician. Psychological support is incorporated into transition strategies; psychologists are included as members of the team or, in some cases, this function is performed by the treating clinician.

### 3.2 Case studies

The following patients have been transitioned to adult care using the above strategies and show potential barriers to transition. A case where transfer to adult care was not considered to be appropriate is also included.

#### 3.2.1 Case 1 – MPS I (Hurler syndrome) with chronic variable immunodeficiency

• Male patient diagnosed at 8 months

##### 3.2.1.1 Barriers to transition

• Symptoms
  o Chronic variable immunodeficiency
  o Respiratory infections (approximately one per year)
  o Severe learning disabilities
  o Severe hearing loss, requiring cochlear implants
  o Corneal clouding
  o Hip dysplasia
• Disease management
  o Bone marrow transplants at 12 and 18 months
  o The immunology team required that immunoglobulin infusions were monitored in a hospital setting once a year from the age of 16
• Patient and family
  o Patient wary of unfamiliar people
  o Parents felt it would be important to meet the adult care team more than once prior to transfer

##### 3.2.1.2 Implementation of transition

• Initiated at age 15, with three visits to the transition clinic over 18 months
  o Early opportunity to establish a relationship with adult care team
  o Extended period to become familiar with adult care setting and changes to consent, and for clinicians to develop follow-up strategies for patient's medical requirements
• Transition passport completed and provided to family, in case of emergency hospital admissions
• Family assured of their continued attendance at adult care appointments
• Patient's immunological needs were managed by a separate immunology team to meet requirements for immunoglobulin infusion monitoring
• At the final transition appointment:
  o Contact details of adult team provided to patient and family
  o ‘Ready Steady Go’ documents completed to ensure patient needs had been recognised and managed prior to transfer
• Transition concluded at 16 years of age, once the patient had no major issues that required resolution

##### 3.2.1.3 Patient outcome

• The patient is currently 20 years of age and remains engaged with adult healthcare services. He regularly receives immunology therapy and has had no recent hospital admissions.

##### 3.2.1.4 Key learnings

• Transfer to adult teams that are responsible for different aspects of symptom and treatment management can be coordinated separately to ensure institutional requirements are met, while allowing the patient sufficient time to transition. For this patient, increased risk of infection and monitoring of infusions were managed by the immunology team.
• Starting the transition process at an early age provides opportunities for patients and families to meet the adult care team and put in place strategies to manage follow-up and emergency care. In this case, the inclusion of the family in appointments in the adult care setting was important, as the patient had learning disabilities and could not fully manage their own health.
• The use of transition documents, such as the ‘Ready Steady Go’ package, allowed the transition team to monitor the patient's readiness for transfer and provided further opportunities to resolve outstanding issues.
• If the patient were to require emergency care with a team that was unfamiliar with his medical needs, the provision of a transition passport means that the patient's parents could easily provide treating HCPs with the relevant information.

#### 3.2.2 Case 2 – MPS I (Hurler syndrome) with severe phenotype, emotional delay and change of hospital

• Female patient diagnosed at 4 years

##### 3.2.2.1 Barriers to transition

• Symptoms
  o Severe phenotype
    ▪ Dysostosis multiplex, short stature, corneal opacity, cardiac valve disease, restrictive lung disease and chronic otitis
• Disease management
  o Patient was referred from another paediatric hospital at age 16; she had a severe phenotype and had not received a bone marrow transplant, so she required a full medical assessment by the specialist paediatric team prior to transition
• Patient and family
  o Patient emotionally immature and wary of changes to environment
  o High dependence on family
  o Family very concerned by potential changes to care in adult setting

##### 3.2.2.2 Implementation of transition

• Initiated at age 18
  o Allowed time for a full medical review after moving from a different hospital
• Introduced to the adult team at an early stage
  o Opportunities to discuss concerns and capabilities of adult care team
• Psychological support provided
  o Support also provided to family
• Transfer at age 19 years

##### 3.2.2.3 Patient outcome

• The patient remained under adult care services until she died from respiratory complications at the age of 22 years.

##### 3.2.2.4 Key learnings

• Starting the transition process early provides opportunities for patients and families to meet the adult care team, and allows sufficient time for HCPs to reassure the patient and their family with regard to key changes they will face in the adult hospital.
• Transition can be delayed if additional time is required to fully assess a patient; for example, if they have moved from another hospital.
• The use of transition checklists allowed the transition team to monitor the patient's readiness for transfer, and provided opportunities for psychological interventions when required for both the patient and family.
  o This checklist supports the MDT in managing parental concerns and expectations that may have a negative impact upon transfer to the adult hospital.

#### 3.2.3 Case 3 – MPS II with complex symptoms

• Male patient diagnosed at 4 years

##### 3.2.3.1 Barriers to transition

• Symptoms
  o Complex symptoms
    ▪ Hearing loss, joint contractures, pain, hepatomegaly, and cardiac and respiratory problems, which required an MDT including multiple types of specialists
• Disease management
  o Joint contractures were particularly challenging to manage because surgical members of the MDT frequently wanted to resolve these through surgery, which was in opposition to other team members
• Patient and family
  o Live at a distant site to the paediatric centre

##### 3.2.3.2 Involvement with adult care system

• Started to attend appointments without parents from the age of 14 years
• Adult care clinician provided psychological support
  o Contact details provided for urgent queries

##### 3.2.3.3 Patient outcome

• The patient now attends appointments at a local centre, but is assessed at an inherited metabolic disease centre twice a year allowing for an expert opinion of all test results and input into care strategies.

##### 3.2.3.4 Key learnings

• Managing complex symptoms in MPS requires input from many different specialists; coordination of such care can be a challenge. Starting the process at an early age allows time for the patient to adjust to their increasing independence and responsibility for their health.
• The provision of psychological support during transition of patients with complex symptoms, especially when not accompanied by parents, allows patients to be reassured throughout the process.
• Having an expert in adult metabolic diseases who can coordinate the members of the MDT is of utmost importance. The role of the coordinating expert is to ensure that MPS-focused input is available after all assessments and when planning medical or surgical procedures.

#### 3.2.4 Case 4 – MPS IIIC with developmental delay

• Female patient diagnosed at 4 years

##### 3.2.4.1 Barriers to transition

• Symptoms
  o Unable to speak; communicated through facial expressions and body language
  o Significant behavioural issues
  o Sleep difficulties
  o Although the patient could still eat orally, she experienced a choking episode, which raised concerns over her ability to swallow
• Disease management
  o Videofluoroscopy assessments ruled out aspiration
  o Thickened foods were recommended, and gastrostomy considered, but the patient's family did not wish the procedure to be carried out because the patient enjoyed participating in family meals
• Patient and family
  o Intermittent engagement with paediatric services, with lapses lasting for up to 6 years
  o Clinical input from a range of team members (physiotherapists, speech and language therapists, occupational therapists, and nurses)
  o Family concerns over lack of information on transition, the involvement of unfamiliar HCPs, post-surgical care and the family's limited involvement in the decision-making process

##### 3.2.4.2 Implementation of transition

• Initiated at age 18
  o Started at a later age because of family's limited engagement with healthcare system
  o Care plan developed and transition passport completed to detail patient needs and family's view that gastrostomy would limit quality of life
• ‘Best interest meeting’ held between paediatric and adult teams to discuss gastrostomy
  o Family's view incorporated into care plan
  o Agreed to manage swallowing difficulties through regular monitoring by a speech therapist
• Opportunities for patient and family to meet the adult care team and be reassured that adult care clinicians would take their views into consideration
• Transfer at age 18 years

##### 3.2.4.3 Patient outcome

• The patient is currently 23 years of age. She has not yet required a gastrostomy and has had no recent hospital admissions. Her family remain her main carers and are involved in making healthcare decisions alongside healthcare professionals.

##### 3.2.4.4 Key learnings

• When patients have developmental delays or communication difficulties, carers have a duty to ensure that patients remain engaged with healthcare services. Where required, parents should be allowed to accompany adult patients to appointments.
• Paediatric teams need to be aware of the services in place in adult care centres to allow patients and families to be informed in advance of likely changes, and for care strategies to be developed. Families also need to be made aware of changes to consent.
• The transition clinic allowed the HCPs involved to focus on the patient's individual needs. Once the family's opinions had been included in the management strategy and they were reassured of the patient's future care, they were happy for the patient to transfer to adult care.
• For this patient, a school nurse and speech therapist were included in the MDT to support her developmental and communication needs and her plans for leaving education.

#### 3.2.5 Case 5 – MPS VI with seizures

• Female patient diagnosed at 5 years

##### 3.2.5.1 Barriers to transition and factors affecting early adult care

• Symptoms
  o Patient had no cognitive deficit but had a low quality of life due to a range of complex symptoms
    ▪ Corneal clouding, joint contractures, growth retardation, obstructive apnoea, hydrocephalus and cardiac dysfunction
  o Patient developed seizures at the age of 21 years, while managed by the adult care team
• Patient and family
  o Patient and family emotionally attached to paediatric team because a lot of time was spent under paediatric care

##### 3.2.5.2 Implementation of transition

• Initiated at age 17
  o Opportunities to meet the adult care team
  o Family reassured of adult care team's capabilities
• Continued input from paediatric specialists when required
  o Paediatric neurologist provided input on seizure management
• Full medical assessment was carried out 2 months before the transfer
• Treating adult care clinician provides MPS-specific support during emergency admissions for seizures, even at other hospitals

##### 3.2.5.3 Patient outcome

• The patient is currently 24 years of age. The family continue to attend infusion appointments and remain involved in the patient's care.

##### 3.2.5.4 Key learnings

• Allowing opportunities for the patient and family to meet the adult care team provides reassurance of the adult care team's skills in managing the patient.
• Even when time for transition is limited, patients can be informed of changes in advance, providing time for patients and families to resolve concerns and familiarise themselves with new settings.
• Inclusion of paediatric and metabolic specialists ensures optimal treatment of patients during emergency situations, in which the treating HCPs may not be familiar with MPS.
• For this patient, neurologists and intensive care clinicians are core team members for seizure management.
  o When the patient experiences a seizure, there is the potential for respiratory arrest, arrhythmia or aspiration; experience of managing these complications in patients with MPS is key to optimal patient outcomes.
• Involvement of the family at infusion appointments helped them to feel reassured over the patient's care.

#### 3.2.6 Case 6 – MPS VI with complex symptoms

• Male patient (age of diagnosis unknown)

##### 3.2.6.1 Barriers to transition

• Symptoms
  o Complex symptoms
    ▪ Recurrent respiratory infections, hearing and vision loss, movement difficulties, growth retardation, foot deformities and hip pain
• Disease management
  o Multiple surgical procedures
    ▪ Multiple spinal decompression surgeries (cervical and thoracolumbar)

##### 3.2.6.2 Involvement with adult care system

• Started to attend appointments without parents from the age of 14 years
• Adult care clinician provided psychological support
  o Contact details provided for urgent queries

##### 3.2.6.3 Patient outcome

• The patient now attends appointments at a local centre but is assessed at an inherited metabolic disease centre twice a year, allowing for an expert opinion of all test results and input into care strategies. He lives in supported accommodation for young people with vision loss. An adult care ophthalmologist, neurosurgeon and ear, nose and throat (ENT), and orthopaedic specialists are included in the MDT.

##### 3.2.6.4 Key learnings

• A competent adult MDT should be in place to enable timely and relevant care for patients with complex symptoms.
• The MDT approach should be individualised to ensure that a patient's specific symptoms are appropriately managed. In this case, an ophthalmologist was a key member of the team because the patient was almost blind. An ENT specialist was also included to manage hearing loss and the use of hearing aids.
• Input from neurosurgeons and orthopaedic specialists has been required to manage decompression surgeries and the negative impact of foot deformities and hip pain.

#### 3.2.7 Case 7 – MPS II who continued under paediatric care

• Male patient diagnosed at 4 years

##### 3.2.7.1 Barriers to transition

• Symptoms
  o Complex symptoms
    ▪ Dysostosis multiplex, cardiac valve disease, hydrocephalus, chronic respiratory insufficiency and severe neurological impairment (tetraplegia, loss of consciousness and seizures)
• Disease management
  o Ventriculoperitoneal shunts to manage hydrocephalus
  o Nocturnal bilevel positive airway pressure
  o Aspiration of secretions
  o Physiotherapy and pulmonary physiotherapy
  o Feeding support

##### 3.2.7.2 Decision to retain patient under paediatric care

• Decision made when patient was 18 years old
• Patient experienced general deterioration of symptoms, and palliative input was required
• Paediatric care team and family involved in decision-making process
  o Strategy presented to the family by the referring clinician
• Retention under paediatric care was considered to be the most appropriate care option because of the paediatric care team's knowledge of the patient's complex symptoms across a range of specialities
  o Family were aware of the patient's prognosis and comfortable with continued management by the paediatric care team based on long-term relationships with individual HCPs, during which many periods of severe or life-threatening disease symptoms were managed
  o The family were concerned that a team of adult specialists would not have such an extensive knowledge of the patient's symptoms as the paediatric team
• The decision required authorisation from the hospital director to allow the patient to be treated in a paediatric emergency setting and admitted to paediatric wards if required

##### 3.2.7.3 Patient outcome

• The patient remained under the care of the paediatric palliative team. He was admitted to hospital with respiratory difficulties on two occasions and died at home at the age of 20.

##### 3.2.7.4 Key learnings

• A patient may remain under paediatric care if their prognosis is poor and the paediatric team have the most appropriate expertise to manage their worsening symptoms.
• Palliative care teams can be incorporated into the MDT to manage later disease stages.
• As for patients who transition to adult care, family involvement should be continued throughout the decision-making process.
• Necessary approvals should be sought to ensure continued, seamless treatment of adult patients within a paediatric setting if this is contrary to hospital or country-specific regulations or guidance.

## 4 Discussion

Through the varied cases presented here, we show that a range of symptoms may need to be considered while managing transition from paediatric to adult care settings in patients with MPS. As expected for severe, complex diseases, each patient presented the paediatric and adult care teams with unique challenges, such as immunodeficiency, respiratory infections, loss of speech and hearing, impaired vision, dysostosis multiplex, cardiac and respiratory dysfunction, hepatomegaly, and pain. For each case, the clinicians modified their centre's procedures to ensure the transition process and transfer were suited to the patient's evolving medical needs and personal preferences. For example, the patient with immunodeficiency and respiratory infections was transferred to different speciality adult care teams at different times, allowing smooth, coordinated management of all aspects of medical care. Indeed, access to multiple specialists should be a core consideration of transition planning. Furthermore, the use of transition documents ensured that all medical information was readily available should an emergency admission be required. For this patient and others with learning disabilities, parental involvement was continued throughout transition and into adult care, providing reassurance for the patient and family. For other patients, additional time may be required to understand their needs, and in these circumstances the transition process can be delayed until the MDT has completed all required medical assessments. As the patients age and continue to be managed by adult care teams, assessment of their medical requirements will continue on a regular basis and the provision of care will be adapted as needed, to ensure that patients with MPS have the best quality of life possible. For some patients who have a poor prognosis or who are receiving palliative care, transfer to an adult care setting may not be in their best interests and, in these circumstances, responsibility may remain with the paediatric care team. Understanding the patient's current and future needs is the key to planning the most suitable management strategy for each individual.

As highlighted through the presented cases, when developing and optimising an effective transition process for patients with severe, progressive symptoms, a number of key factors should be considered to bridge the gap between paediatric and adult care and to ensure patients remain effectively managed in healthcare systems.

### 4.1 Early planning

The transition process should start in early adolescence, allowing sufficient time for the patient and family to come to terms with the changes ahead [2,[4], [5], [6], [7],^11^]. This provides the opportunity for patients and carers to develop relationships with the adult care team, familiarise themselves with the facilities and routines in the new setting, and be reassured of the new team members' capabilities. For patients who may remain under paediatric care, early planning allows time to request any relevant approvals that ensure the patient's continued access to treatment in this setting.

### 4.2 Education and engagement

The use of educational resources for both patients and families should be coordinated within the process, in order to guide the patient to a point at which they can manage their own medical care, if they are capable [2,[4], [5], [6],^15^]. Introducing carers to the purpose of transition and its importance to the patient's health can ensure that they are supportive in advance of the implementation of practical changes to clinical care. Practical information on differences between paediatric and adult care settings is also invaluable in ensuring everybody involved feels more at ease with the move to the adult care system. Each patient should receive the relevant information in a form that is easily understandable and individualised for their specific needs and queries.

### 4.3 Transition documentation

The use of transition documents and checklists allows the transition team to monitor the patient's readiness for transfer and anticipate challenges ahead, and provides further opportunities to resolve outstanding issues [2,4,7]. This monitoring can be used to prompt psychological input in patients requiring additional support, which can be extended to family members too. Before a patient moves to the adult care setting, a summary or ‘passport’ of their medical history, requirements and preferences should be prepared so that each member of the adult care MDT or emergency team has access to all relevant information. If the patient were to require emergency care with a team unfamiliar with their medical needs, the provision of a transition passport can provide treating HCPs with easily accessible, relevant information [2,4].

### 4.4 Clinical expertise

While the principles of successful transition can be transferred from experiences in other chronic paediatric diseases, one of the difficulties is the limited experience of rare paediatric diseases in adult healthcare systems. This can result in reduced patient, carer and paediatrician confidence in disease management capabilities once the patient has moved to the adult care team [3]. Starting the transition process early allows time for an adult care team with expertise in MPS to be assembled (if one is not already in place) and medical plans and emergency strategies to be developed [15]. Adult care teams can remain in contact with paediatricians even after patient transfer [2,4]. Indeed, transfer need not mean the end of the involvement of paediatric experts, who can continue to provide support and a wealth of information on the intricacies of managing complex rare diseases [2]. While this support could take the form of advice or training, it may also include access to paediatric surgical equipment that is not routinely used by the adult care team. For example, surgery in patients with MPS of short stature may require the use of small endotracheal tubes [38,47] or small replacement cardiac valves, both of which are available to paediatric surgeons. The continued involvement of the paediatric team allows surgical preparations in the adult care setting to be optimised and individualised, even if non-standard equipment and advice on infrequently used procedures are needed. For some patients with advanced severe disease and a poor prognosis, the most appropriate option may be to continue under paediatric care with HCPs who are familiar with the patient's complex medical history and current symptoms.

### 4.5 Coordination

The MDT needs to be coordinated throughout the transition process, with input from paediatric and adult care teams as appropriate [2,4,9,15,48,49]. The potentially large number of transition team members and the challenging assessments across multiple organs requires a member of the team with practical experience of MPS disorders to be accountable for coordinating the care of individual patients [7]. Although non-clinical transition coordinators can also be included as members of the team in order to direct patients towards patient organisations and other supportive resources, a clinician with experience of MPS should always be a core team member.

### 4.6 Family involvement

As the transition period is a change for the family as well as the patient, family involvement should be continued when possible, if the patient agrees. In some cases, such as for patients with learning or communication difficulties, family members have a duty of care and their continued input is required. Allowing input from parents and carers, while ensuring that the patient has opportunities to discuss their own feelings and develop independence, can ensure all parties are supportive of the transition process.

### 4.7 Flexibility and individualisation

Each patient manifests with variable symptoms, rates of progression, capabilities and interactions with their carers. While structured transition processes are required, these plans must be individualised for each patient and remain flexible throughout to ensure that, as each patient moves through adolescence, changes in their independence and support needs are reflected in their progress towards disease self-management and adult care [7]. The adult care MDT should also be individualised to each patient's MPS complications and this can be planned during transition. When a patient is not able to be transferred to adult care (for example, if they are receiving palliative care or have complex surgery planned in a paediatric setting), systems can be put in place to allow them to remain under paediatric care. The start of transition can be delayed if additional time is required to fully assess a patient if, for example, they have moved from another hospital [9,15]. As patients transfer to adult care, each transition experience can be assessed to identify areas where changes to the strategies are needed. These evaluations, for example, may identify transition documents that are not clearly understood by patients and families, or stages of the process that require more time. Requirements for patients with particular symptoms and clinical needs can be recorded, and used to inform individualisation of strategies for future patients. For example, with regard to the patient with chronic variable immunodeficiency (Case 1), an understanding of how the immunology team would manage the patient's immunoglobulin infusions could be noted and use to plan transition strategies for future patients requiring this treatment.

In patients with lysosomal storage disorders, such as MPS, other disease-specific measures may need to be incorporated into the transition strategy, such as establishing a home care infusion routine, ensuring mental capacity assessments are up to date and planning a disease monitoring scheme that provides appropriate surveillance of any potential adult-onset symptoms [2]. Patients with MPS may also experience symptoms associated with low body weight or short stature, with some patients reaching a height of less than one metre or even experiencing decreases in height over time [35,37,40]. These symptoms require attention, as they may result in patients needing lower doses of particular treatments, for example, during anaesthesia, or paediatric equipment during surgery. Indeed, transition teams have a key role to play in ensuring that adult care teams are aware of, and prepared for, managing factors that are not frequently seen in an adult population. Weight and height may have a further negative impact on practical aspects of daily living, such as reaching light switches and operating door handles, and small patients may also be more prone to being treated as children, with healthcare professionals interacting with family and carers, rather than directly with the patient. These practical and social factors must also be considered in the transition plan, to ensure that all aspects of the patient's life that are affected by MPS symptoms are managed as fully as possible.

### 4.8 Conclusion

While this publication is focused on patients with MPS, the strategies adopted by the contributing centres are shared across other studies of transition and incorporate an individualised approach for each patient. By employing strategies that are coordinated and individualised, start early, and provide information throughout for patients and carers while remaining flexible, centres that manage patients with MPS can enhance a patient's chances of a smooth transfer from paediatric to adult care. The cases presented in this paper exemplify the challenges that may be encountered when transitioning patients with MPS and the practical steps that can be incorporated into transition management processes to mitigate these difficulties and support continued disease management.

## Declaration of Competing Interest

CL report honoraria from BioMarin Pharmaceutical Inc., Shire/Takeda and Sanofi Genzyme, Alexion Pharmaceutical, Ultragenyx and Chiesi Farmaceutici outside of the scope of this project. BM has received honoraria from BioMarin Pharmaceutical Inc., and from Sobi outside of the scope of this project. CH owns a company that performs contractual work with BioMarin Pharmaceutical Inc. outside of the scope of this manuscript. MdT has received honoraria from BioMarin Pharmaceutical Inc. AG, TVL, JPL, KMS and NDV have no conflicts to report.

## Appendix A Supplementary data

Supplementary material Image 1

Barcelona checklist_formatted Image 2

Salford Capacity leaflet Image 3

Salford Go Image 4

Salford Parent_carers leaflet Image 5

Salford Ready Image 6

Salford Steady Image 7

Salford Transition leaflet Image 8

Salford Transition plan Image 9
