# Supplementary material for: Transition of patients with mucopolysaccharidosis from paediatric to adult care
Source: Mol Genet Metab Rep. 2019 Oct 21;21:100508. doi: 10.1016/j.ymgmr.2019.100508 (PMC6819742; doi:10.1016/j.ymgmr.2019.100508)
Supplement: Supplementary file 1 — Supplementary material [file mmc1.docx]

# Supplementary materials

## Centre-specific transition information

### The Mark Holland Metabolic Unit, Salford Royal NHS Foundation Trust, Salford, UK

- Transition documents

### Research Center for Children’s Health, Moscow, Russia

- City Clinical Hospital V.V. Vinogradov website – http://gkb64.ru/
- National Medical Research Center for Children’s Health website – http://nczd.ru/

### Vall d’Hebron University Hospital, Barcelona, Spain

- Transition checklist

### HELIOS Dr. Horst Schmidt Kliniken, Wiesbaden, Germany

- HELIOS Dr. Horst Schmidt Kliniken website – https://www.helios-gesundheit.de/kliniken/wiesbaden-hsk/
- Society for Mucopolysaccharidosis website – https://mps-ev.de/mps/

## Transition strategies schematics

### 1 The Mark Holland Metabolic Unit, Salford Royal NHS Foundation Trust, Salford, UK

This centre manages patients with a range of rare metabolic disorders, including MPS. In the UK, patients under the age of 16 years cannot be managed by an adult team, while patients over the age of 18 years cannot be managed by a paediatric team. From the age of 16, patients in the UK provide their own consent for medical treatment, unless they do not have capacity to manage their own care [1]. In these cases, the Mental Capacity Act 2005 will be adhered to [2]. In some exceptional cases (for example, patients under palliative care), responsibility for patients aged over 18 years may remain with a paediatric team. The transition strategy at this centre is aligned with the National Institute for Health and Care Excellence (NICE) guidelines on ‘Transition from children’s to adults’ services for young people using health or social care services’ (NG43) [3]. These guidelines define the responsibilities and processes that should be in place to guide individuals from paediatric to adult care services, and focus on developmentally appropriate, individualised strategies that provide an integrated transition, coordinated by a named individual. They emphasise that the patient should be an equal partner in decision-making throughout the process.

The centre’s transition strategy has been developed over recent years as increasing numbers of patients survive into adulthood. To guide patients through transition, the team at the Mark Holland Metabolic Unit uses a series of documents, including ‘Ready Steady Go’ to monitor the patient’s readiness to transfer [4] and a transition passport that explains transition and records the patient’s medical details and preferences on communication (including who they would like to be involved in the transition). The transition passport can be retained by the transition clinic to ensure it is available at every patient visit.

The transition process usually commences at the age of 16–17 years, although the centre is aiming to reduce this to 14–15 years and to begin education on the transition process at the age of 12 years. The transition process occurs over approximately two years to allow time for the family to engage with the adult team and understand the differences between paediatric and adult care systems, such as the greater patient responsibility and more formal setting when under adult care. Paediatricians provide the adult care teams with a list of patients who are at an appropriate age to start transition and circulate referral letters for the transition clinic. At the patient’s first visit to the transition clinic, they will participate in a 1:1 meeting with a metabolic nurse to discuss their understanding of their disease and their role in managing their health. The transition passport and ‘Ready Steady Go’ documents are introduced at this point. The patient then visits the transition clinic every 6 months until they are ready to transfer to adult care, as assessed through ‘Ready Steady Go’. A parent/carer assessment is also included as part of the ‘Ready Steady Go’ materials, allowing parents and carers to understand what they will need to do when their child transfers to adult care and the responsibilities their child will take on. Leaflets are also provided to summarise the transition process and why it is needed, and to explain what will happen if the patient lacks the mental capacity to make decisions concerning their healthcare. The transition clinic includes input from paediatric and adult care HCPs, including metabolic consultants, a learning disabilities nurse, an adult metabolic specialist nurse, an adult metabolic dietician and a physiotherapist. The clinic is held up to twice a month at different sites, to allow flexibility for attending patients, and is managed by an administrative team who coordinate transfer of medical details between HCPs. Queries during transition are managed by the paediatric team, with input from the adult care team when required. During transition, infusions remain under the management of home care nurses, who arrange transfer of therapy between paediatric and adult teams.

Patients usually transfer fully to adult care between the ages of 16 and 18 years. At this time, a detailed clinical summary of medical and surgical procedures, medical requirements, assessments, and prescriptions is sent from the paediatric team to the adult metabolic team. The overall time spent in transition depends on comorbidities, such as neurological deterioration, as well as hospital admissions, newly emerging symptoms and the patient’s ability to manage their health. The overall transition process for the Mark Holland Metabolic Unit is shown in Supplementary Figure 1. All patients with MPS (twelve in total: five with MPS I, four with MPS III and three with MPS IV) who have transitioned since 2016 currently remain under routine adult care.


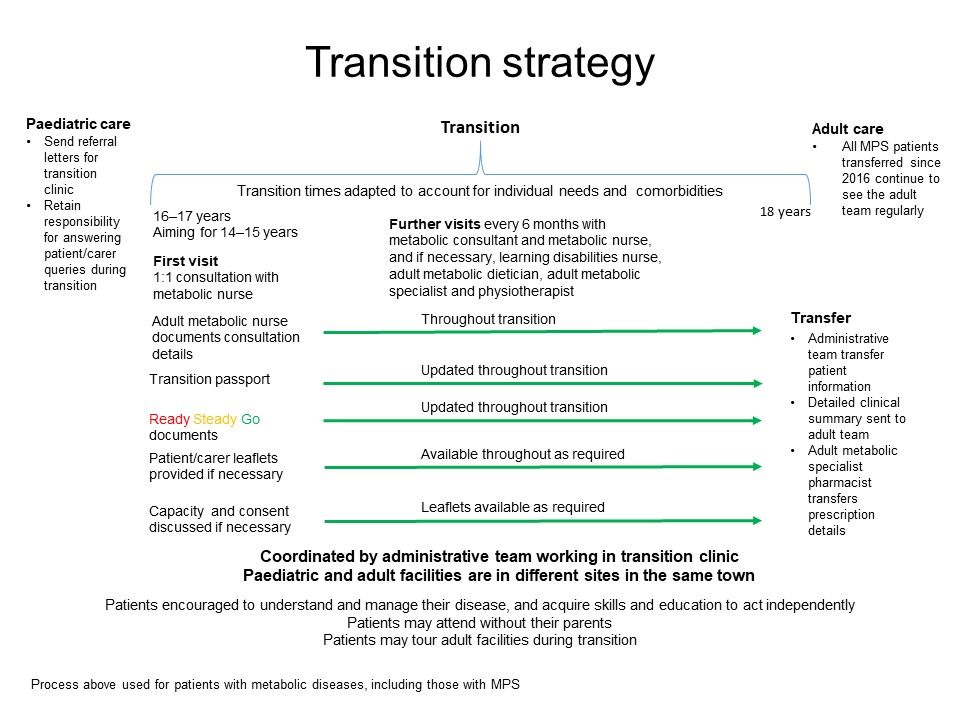


**Supplementary Figure 1. Summary of transition strategy at the Mark Holland Metabolic Unit, Salford Royal NHS Foundation Trust, UK**

### 2 Research Center for Children’s Health, Moscow, Russia

Russian regulations mean that patients over the age of 18 years must be managed by adult care HCPs, while all patients under this age are treated solely by paediatric teams. Russian guidelines for the management of patients with MPS are based on international publications [5, 6]. The transition process is coordinated by the paediatric team and used to manage a range of metabolic diseases, including MPS. When patients reach the age of 17 years, their paediatrician contacts the adult care team, and at this stage the patient has opportunities to meet the adult team and tour the facilities, which are based at a different site to the paediatric team. This transition visit is carried out as part of the patient’s 6-monthly visit schedule. A second visit is carried out prior to transfer to adult care, and a full medical examination is conducted in paediatrics to ensure that a detailed record of the patient’s medical requirements is provided to the adult care team. Information on the transition process for patients and carers is provided through websites, details of which are passed to patients by their HCPs. Queries on the transition process are managed by the paediatric team, and psychologists are available to provide additional support if required. At the age of 18, patients are formally transferred to the adult care team over a period of approximately two weeks, during which time infusions may be carried out at either the paediatric or the adult centre. Patient organisations may be able to assist with transport plans for patients to attend clinics at a different site. After transfer, the adult care team may be supported by the paediatric team if further guidance is needed to manage a particular patient’s disease. The transition process used at the Research Centre for Children’s Health is shown in Supplementary Figure 2. Although the transition process is often well managed in large cities (such as Moscow and Saint Petersburg), in smaller regions the hospitals may have less experience of adult patients with MPS, and patients may travel long distances each week for routine follow-up appointments and enzyme replacement therapy. Since 2016, three patients with MPS (subtypes I, II and VI) have been transferred from paediatric to adult care at this centre.


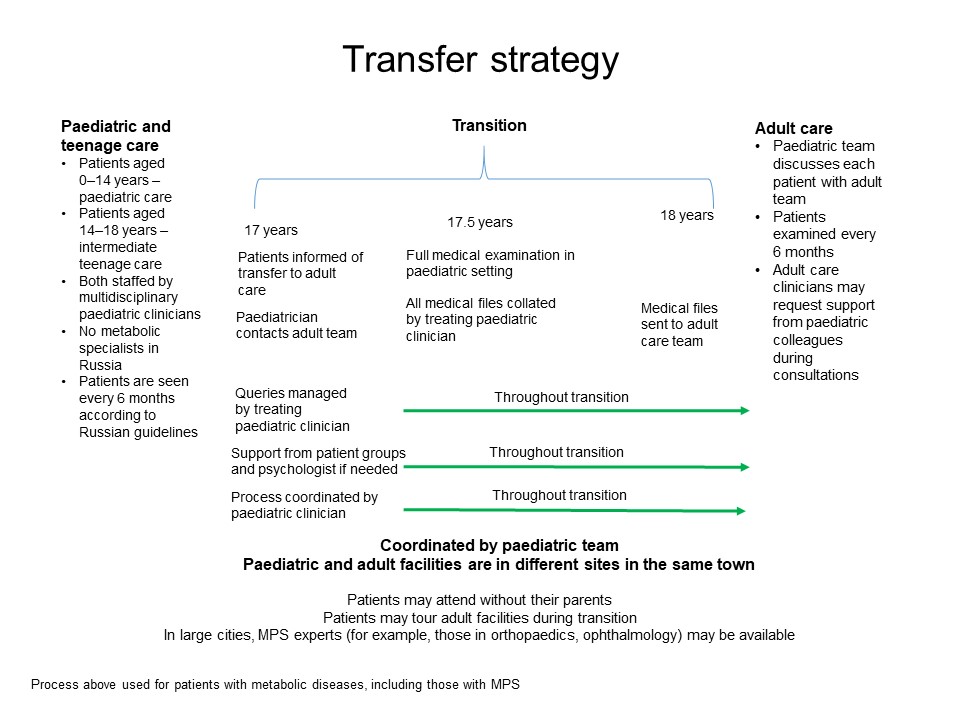


**Supplementary Figure 2. Summary of transition strategy at the Research Center for Children’s Health, Russia**

### 3 Vall d’Hebron University Hospital, Barcelona, Spain

In some regions of Spain, patients over the age of 16 should not be admitted to paediatric emergency departments, although in other regions patients may attend up to the age of 18 years. Outpatient paediatric clinics may see patients up to the age of 18, or 20 in exceptional circumstances (such as neurological deterioration, short life expectancy or planned surgery). The transition process is coordinated by paediatric and adult care metabolic HCPs. At the age of 16, patients are referred to an adult care team, and their clinical history is transferred by the paediatric metabolic team if they do not have any active symptoms that are undergoing current management by the paediatric team. An initial visit is held with an adult metabolic HCP, and patients then attend a transition visit every 4–6 months, without their parents if they wish. At the initial visit, the need for transfer is explained, and the HCP confirms the patient’s understanding of their disease and their future needs. One or two transition visits are held with input from both paediatric and adult care teams, and an MDT meeting is held to evaluate the severity of symptoms and organise sequential transfer to each adult specialist based on the patient’s medical requirements at the time. Psychological support is available, if required. Information on the new adult care setting is provided and a checklist is used to monitor the patient’s progress through transition. The paediatric team manages any patient queries during transition. Paediatric and adult care nurses coordinate the transfer of medical records when the patient moves to adult care. Time spent in transition varies between 1 and 2 years to take into account the need for the management of comorbidities and planned surgeries. After transfer, family members may still attend appointments if patients lack capacity to make decisions regarding MPS management. The overall process is shown in Supplementary Figure 3. Seven patients have transferred since 2016; one patient with each of MPS I, MPS II, MPS IIIA, MPS IIIB, MPS VII, and two with MPS IV.


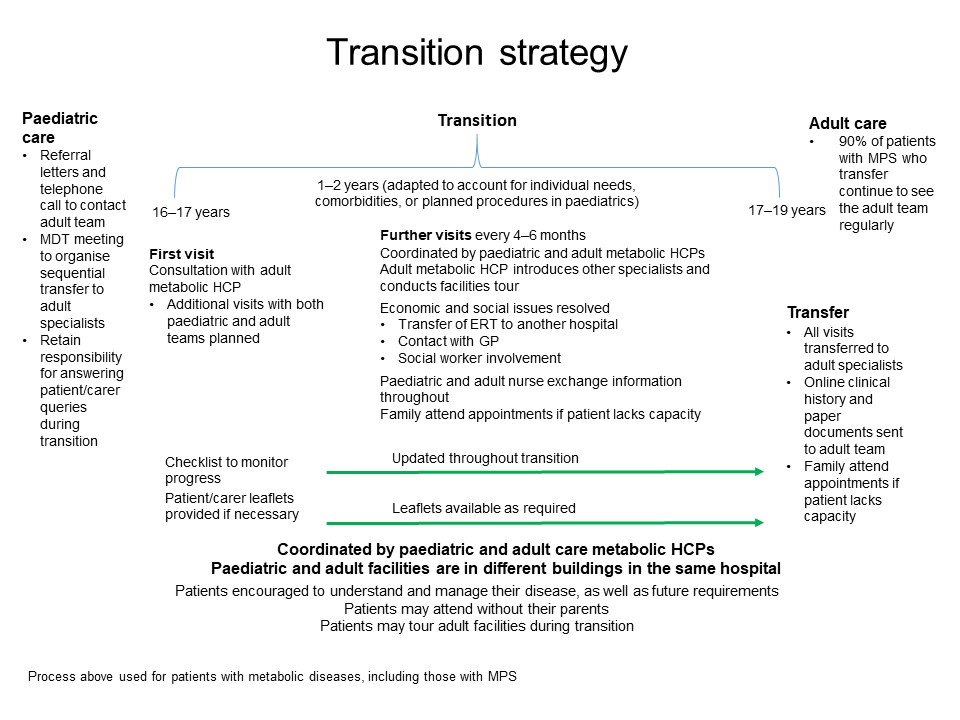


**Supplementary Figure 3. Summary of transition strategy at Vall d’Hebron University Hospital, Spain**

ERT, enzyme replacement therapy; GP, general practitioner; HCP, healthcare professional; MDT, multidisciplinary team.

### 4 HELIOS Dr. Horst Schmidt Kliniken, Wiesbaden, Germany

In Germany, adult care clinicians may treat paediatric patients, but paediatricians are usually not reimbursed for the treatment of adult patients. Furthermore, adult patients may stay in paediatric inpatient wards. At this centre, all patients with MPS officially remain under paediatric care, although an adult care clinician gradually assumes responsibility for planning patient care and recruits input from other adult care specialists as required.

As such, there is no formal transition process or transfer of patients with MPS. The paediatrician and adult care clinician act as a coordination point for the input of members of the MDT, and discuss the need for medical and surgical interventions, with input from the patient. Although officially under paediatric care, patients are able to attend some parts of their appointments with the adult care clinician alone from the age of 14, and input from adult care specialists is sought as required. From the age of 16, full appointments may be carried out without parental attendance. Information on MPS and the adult care setting is provided through websites, and care plans are tailored for each patient’s capabilities. The overall process is shown in Supplementary Figure 4. Since 2016, 26 patients between the ages of 14 and 18 have been the responsibility of the adult care clinician (five MPS I, four MPS II, six MPS III, five MPS IV and six MPS VI).

This centre uses a different strategy to the others, as responsibility for the management of care plans remains within the same setting. Generally, it has been found that patients and families at this centre do not wish to move to an adult care setting, and this strategy means that they remain in the same, familiar setting, with input from adult care specialists sought as required. While this strategy is in place because of the limited numbers of clinicians with experience in MPS, it does provide continuity of care as patients age. The adult care specialist also provides psychological support for the patient, allowing continuity of guidance as well as long-term oversight of medical needs. This management of adult patients within a paediatric setting resolves issues surrounding a lack of adult care HCPs with expertise in MPS. By maintaining contact with the MDT, the centre can ensure that all relevant information is passed on prior to surgery or changes to medical care to ensure that the complex clinical presentations of MPS disorders are accounted for.


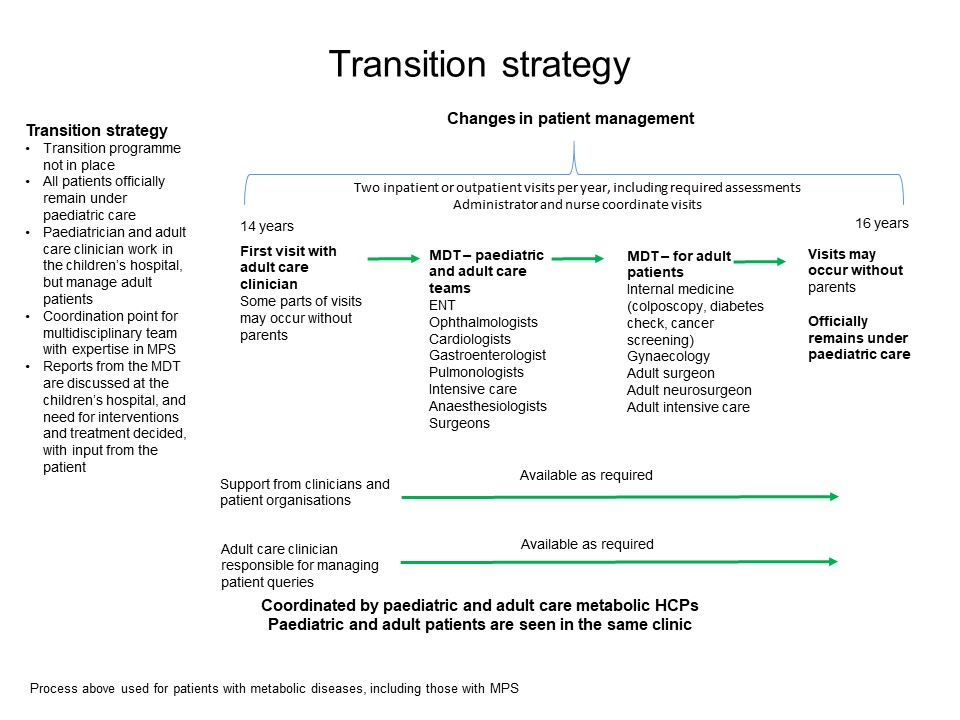


**Supplementary Figure 4. Summary of transition strategy at HELIOS Dr. Horst Schmidt Kliniken, Germany**

ENT, ear, nose and throat; HCP, healthcare professional; MDT, multidisciplinary team.

## References

[1] General Medical Council. Definitions of children, young people and parents, 2018.

[2] Mental Capacity Act 2005. Available at: <http://www.legislation.gov.uk/ukpga/2005/9/pdfs/ukpga_20050009_en.pdf>, Accessed June 2019.

[3] National Institute for Health and Care Excellence, Transition from children’s to adults’ services for young people using health or social care services. NICE guideline (NG43); February 2016.

[4] A. Nagra, P.M. McGinnity, N. Davis, A.P. Salmon, Implementing transition: Ready Steady Go, Arch Dis Child Educ Pract Ed 100 (2015) 313–320.

[5] R. Giugliani, P. Harmatz, J.E. Wraith, Management guidelines for mucopolysaccharidosis VI, Pediatrics 120 (2007) 405–418.

[6] C.J. Hendriksz, K.I. Berger, R. Giugliani, P. Harmatz, C. Kampmann, W.G. Mackenzie, J. Raiman, M.S. Villarreal, R. Savarirayan, International guidelines for the management and treatment of Morquio A syndrome, Am. J. Med. Genet. A 167A (2015) 11–25, https://doi.org/ 10.1002/ajmg.a.36833.
