# Supplementary material for: Transition of patients with mucopolysaccharidosis from paediatric to adult care
Source: Mol Genet Metab Rep. 2019 Oct 21;21:100508. doi: 10.1016/j.ymgmr.2019.100508 (PMC6819742; doi:10.1016/j.ymgmr.2019.100508)
Supplement: Supplementary file 2 — Barcelona checklist_formatted [file mmc2.docx]

TRANSITION PROCESS CHECK LIST

Vall d’Hebron University Hospital, Barcelona, Spain

PLANNING (Year before)

Multidisciplinary team meeting

Active problems

Family informed

Patient informed

Patient understands his/her condition

PREPARATION

Identification and contact with the adult physician

List of problems and needs to cover

Treatment

Specialists

Contact with other professionals

Nurse

Social worker

GP

Reference hospital

Information updated and prepared

IMPLEMENTATION

Visits with both teams (1 to 3)

Visit alone with adult team

Discharge from paediatric centre
