# Supplementary material for: Transition of patients with mucopolysaccharidosis from paediatric to adult care
Source: Mol Genet Metab Rep. 2019 Oct 21;21:100508. doi: 10.1016/j.ymgmr.2019.100508 (PMC6819742; doi:10.1016/j.ymgmr.2019.100508)
Supplement: Supplementary file 3 — Salford Capacity leaflet [file mmc3.pdf]

What to expect from a best interest meeting?

Due to the nature of the meeting there will be some difficult discussions that take place and we would expect you to find it emotional.

Therefore, we would advise you to consider these decisions prior to the meeting and to attend with some views about the decisions. Additionally, we have access to a counselling service within the department and you may find it useful to engage with them prior to the meeting.

Mental Capacity Act 2005

The above information is covered by the Mental Capacity Act 2005, which is a legal framework that should be utilised by all healthcare providers.

It is a legal requirement that the act is applied from the age of 16, as this is the age that a young adult can legally consent for themselves.

Where do I get more information from?

You can discuss this further with your nurse or doctor. You can also visit the following website:

https://www.justice.gov.uk/protecting-the-vulnerable/mental-capacity-act

© G15030505W. Design Services
Salford Royal NHS Foundation Trust
All Rights Reserved 2015
This document **MUST NOT** be photocopied

Information Leaflet Control Policy:
Unique Identifier: MED07(15)
Review Date: March 2017

Salford Royal NHS
NHS Foundation Trust
University Teaching Trust

safe • clean • personal

For further information on this leaflet, it's references and sources used, please contact **0161 206 4365.**

Copies of this information are available in other languages and formats upon request.
In accordance with the Equality Act we will make 'reasonable adjustments' to enable individuals with disabilities, to access this treatment / service.

If you need this interpreting please telephone

Polish
Jeżeli potrzebne jest Państwu to tłumaczenie, proszę zadzwonić pod numer.

Urdu
اگرآپ کواس ترجمانی کی ضرورت ہے تو مہربانی کرکے فون کریں۔

Arabic
اذا كنتم بحاجة الى تفسير او ترجمة هذا الرجاء الاتصال

Chinese
如果需要翻译，请拨打电话

Farsi
اگر به ترجمه این نیاز دارید ، لطفاً تلفن کنید

0161 206 0224

Email: InterpretationandTrans@srft.nhs.uk

Salford Royal operates a smoke-free policy.

For advice on stopping smoking contact the Hospital Specialist Stop Smoking Service on 0161 206 1779

Salford Royal NHS Foundation Trust<br>Stott Lane,<br>Salford,<br>Manchester,<br>M6 8HD<br>**Telephone**<br>**0161 789 7373**<br>***www.srft.nhs.uk***	If you would like to become a Foundation Trust Member please visit:<br>***www.srft.nhs.uk/for-members***	If you have any suggestions as to how this document could be improved in the future then please visit:<br>***http://www.srft.nhs.uk/for-patients***

Salford Royal NHS
NHS Foundation Trust
University Teaching Trust

safe • clean • personal

Capacity

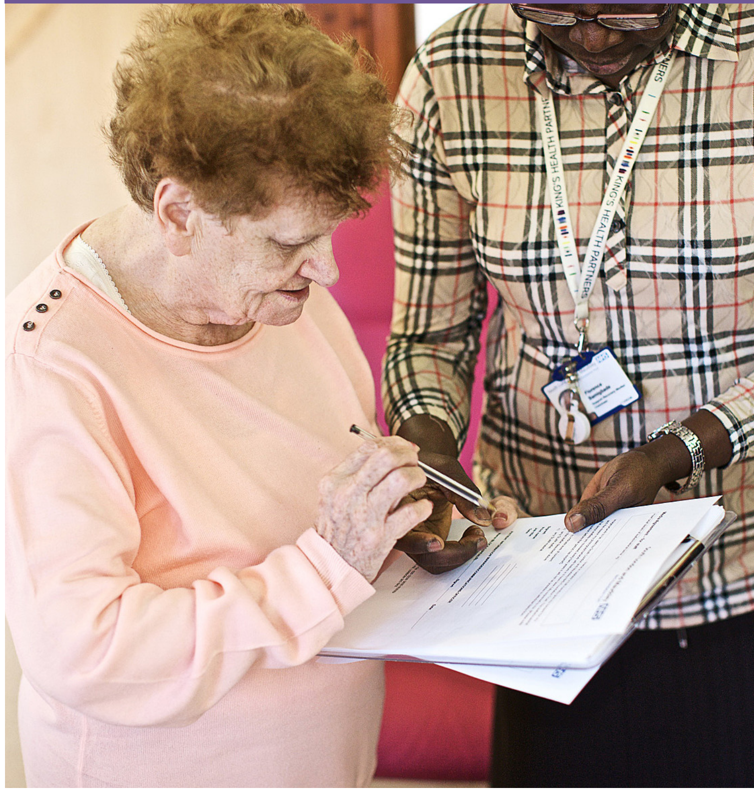

Ladywell Building
Metabolic Medicine
0161 206 4365

Health & care information you can trust
The Information Standard Certified Member

What is capacity?

Capacity is the ability to make an informed decision, this involves understanding the information provided, retaining this information, weighing the options in order to make a decision and be able to communicate the decision.

Why is capacity assessed?

When a decision regarding your care is required and there is a reason to doubt capacity; this could be because of an impairment of, or a disturbance in the functioning of, the mind or brain.

When do you assess capacity?

Capacity would need to be assessed when a specific decision needs to be made regarding your medical treatment/care.

How do you assess capacity?

A healthcare professional would carry out the assessment during your clinic appointment or on a home visit.

The assessment would include individual questions being asked regarding the specific decision; this will involve the use of pictures, body language, gestures and other communication devices that you may use.

What happens if you do have capacity?

If you show that you have the capacity to make this decision then we would provide you with the appropriate support to do this.

At present you may have capacity but due to your condition this could change in the future and you may want to talk to someone about making advance decisions about your care.

What happens if you don't have capacity?

If you have an advance decision statement then this is a legal document, which will enable the care provider to follow the treatment plan based on this decision. If a lasting power of attorney is in place then they will make this decision on your behalf.

When an advance decision statement or lasting power of attorney is not in place, then a best interest meeting is needed in order to make this decision.

What is a best interest meeting?

A best interest meeting is carried out with all of those involved in your care to discuss the treatment/care that is required in order for an agreed decision to be made. This decision will be in your best interest and will be personalised to ensure that the appropriate decision for you is made.

This meeting will take place at Salford Royal so that your specialist metabolic team are involved in the decisions, as they will be able to provide information regarding your metabolic condition.

There will be a decision maker who is usually your consultant and this person will chair the meeting and ensure that the focus of the meeting remains on the individual. A member of the metabolic team will organise this to ensure that all the relevant professionals and family members are invited to the meeting.

If a professional/family member is unable to attend, then we ask that they provide their input prior to the meeting so that their views and wishes can be included. As a result of this, a best interest document will be produced stating the specific decisions made and this will be circulated to all of those involved in your care.

© G15030505W. Design Services, Salford Royal NHS Foundation Trust,
All Rights Reserved 2015. Document for issue as handout.
Unique Identifier: MED07(15). Review date: March 2017
