# Supplementary material for: Transition of patients with mucopolysaccharidosis from paediatric to adult care
Source: Mol Genet Metab Rep. 2019 Oct 21;21:100508. doi: 10.1016/j.ymgmr.2019.100508 (PMC6819742; doi:10.1016/j.ymgmr.2019.100508)
Supplement: Supplementary file 5 — Salford Parent_carers leaflet [file mmc5.pdf]

Parent/carer’s transition plan

Ready Steady Go programme

Name of parent:

Name of patient:

Date: Review (1) Review (2) Review (3)

Internet access: YES/NO

This transition plan is designed to help parents and carers feel confident about their knowledge and skills during the period of transition. Over the next few years we aim to equip your son/daughter and you with the necessary skills to manage their condition and hopefully increase your confidence and that of your son/daughter to transfer to adult services

.

Knowledge and skills

	Yes	No	N/A
I understand the meaning of transition	☐	☐	☐
I know who’s in the team and their respective roles	☐	☐	☐
I know about resources that offer support for parents/carers of young people with my son/daughter’s condition.	☐	☐	☐
I understand what is likely to happen in the future regarding my son/daughter’s condition.	☐	☐	☐
I understand the changes (physical and emotional) which occur during adolescence and how their condition potentially affects and is affected by this development.	☐	☐	☐
I am confident my son/daughter is knowledgeable about their condition and its therapy.	☐	☐	☐
I encourage my child to regularly exercise. I am aware of any restrictions my son/daughter may have.	☐	☐	☐
I feel confident in teaching my son/daughter to become responsible for their own medication at home.	☐	☐	☐

Parent/carers transition plan

Encouraging independence

	Yes	No	N/A
Is your son/daughter independent at home - dressing, bathing, preparing meals, doing chores, etc?	☐	☐	☐
I feel confident for my son/daughter to be seen on their own in clinic for part or all of clinic visits	☐	☐	☐
I understand my son/daughter's rights to information, privacy and confidentiality	☐	☐	☐
I know how to advise my son/daughter about financial help and other support	☐	☐	☐

Healthy lifestyle

I understand the importance of an appropriate healthy diet for young people	☐	☐	☐
I understand the effect of smoking, drugs and/or alcohol on my son/daughter's condition and general health	☐	☐	☐
I know where to access reliable sexual health information for young people and their parents	☐	☐	☐

Preparation for adult services

I feel confident in teaching my son/daughter how to contact the hospital themselves and to organise their repeat prescriptions	☐	☐	☐
I understand the differences between paediatric and adult medical care	☐	☐	☐
I know the plan for my son/daughter's medical care when he/she is an adult	☐	☐	☐

Please list any other things you have concerns about or would like extra help/advice with:

Thank you

Copyright @ Southampton Children's Hospital, University Hospitals Southampton NHS Foundation Trust 2014. All rights reserved. Users are permitted to use Ready Steady Go and Hello to Adult Services materials in their original format purely for non-commercial purposes. No modifications or changes of any kind are allowed without permission of the copyright holder. The following acknowledgement statement must be included in all publications which make reference to the use of these materials: "Ready Steady Go and Hello to Adult Services developed by the Transition Steering Group led by Dr Arvind Nagra, paediatric nephrologist and clinical lead for transitional care at Southampton Children's Hospital, University Hospitals Southampton NHS Foundation Trust."

Ready Steady Go is based on the work of: 1. S Whitehouse and MC Paone. Contemporary Paediatrics; 1998, 13-16. 2. Paone MC, Wigle M, Saewyc E. Prog Transplant 2006; 16:291-302. 3. Janet E McDonagh et al, J Child Health 2006; 10(1):22-43. Further information at www.uhs.nhs.uk/readysteadygo
