# Supplementary material for: Transition of patients with mucopolysaccharidosis from paediatric to adult care
Source: Mol Genet Metab Rep. 2019 Oct 21;21:100508. doi: 10.1016/j.ymgmr.2019.100508 (PMC6819742; doi:10.1016/j.ymgmr.2019.100508)
Supplement: Supplementary file 6 — Salford Ready [file mmc6.pdf]

The Ready Steady Go transition plan - Getting Ready

Ready Steady Go programme

The medical and nursing team aim to support you as you grow up and help you gradually develop the confidence and skills to take charge of your own healthcare.

Filling in this questionnaire will help the team create a programme to suit you.
Please answer all questions that are relevant to you and ask if you are unsure.

Name: Date:

Knowledge and skills	Yes	I would like some extra advice/help with this	Comment
KNOWLEDGE			
I can describe my condition			
I know when to take my medications, names, doses, how often, etc			
I know who's who in the medical and nursing team.			
I understand the differences between paediatric and adult health care			
I know about resources that offer support for young people with my condition			
SELF ADVOCACY (speaking up for yourself)			
I feel ready to start preparing to be seen alone for part of the clinic visit in the future			
I ask my own questions in clinic			
HEALTH AND LIFESTYLE			
I understand it is important to exercise for my general health and condition			
I understand the risks of alcohol, drugs and smoking to my health			
I understand what appropriate eating means for my general health			
I am aware that my condition can affect how I develop e.g. puberty			
I know where and how I can access information about sexual health			

The Ready Steady Go transition plan - Getting Ready

Knowledge and skills	Yes	I would like some extra advice/help with this	Comment
DAILY LIVING			
I can look after myself at home in terms of dressing and bathing/showering etc			
I can make my own snacks/meals			
I am able to be away from home overnight.			
SCHOOL AND YOUR FUTURE			
I am managing at school e.g. getting to and around school, school work, PE, friends, etc			
I know what I want to do when I leave school			
LEISURE			
I can use public transport and access my local community, e.g. shops, leisure centre, cinema			
I see my friends outside school hours			
MANAGING YOUR EMOTIONS			
I know how to deal with unwelcome comments/ bullying			
I know someone I can talk to when I feel sad/ fed-up			
I know how to deal with emotions such as anger or anxiety			
I am happy with life			
TRANSFER TO ADULT CARE			
I understand the meaning of 'transition' and transfer of information about me			

Please list anything else you would like help or advice with:

Thank you

Copyright @ Southampton Children's Hospital, University Hospitals Southampton NHS Foundation Trust 2014. All rights reserved. Users are permitted to use Ready Steady Go and Hello to Adult Services materials in their original format purely for non-commercial purposes. No modifications or changes of any kind are allowed without permission of the copyright holder. The following acknowledgement statement must be included in all publications which make reference to the use of these materials: "Ready Steady Go and Hello to Adult Services developed by the Transition Steering Group led by Dr Arvind Nagra, paediatric nephrologist and clinical lead for transitional care at Southampton Children's Hospital, University Hospitals Southampton NHS Foundation Trust."

Ready Steady Go is based on the work of: 1. S Whitehouse and MC Paone. Contemporary Paediatrics; 1998, 13-16. 2. Paone MC, Wigle M, Saewyc E. Prog Transplant 2006; 16:291-302. 3. Janet E McDonagh et al, J Child Health 2006; 10(1):22-43. Further information at www.uhs.nhs.uk/readysteadygo
