# Supplementary material for: Transition of patients with mucopolysaccharidosis from paediatric to adult care
Source: Mol Genet Metab Rep. 2019 Oct 21;21:100508. doi: 10.1016/j.ymgmr.2019.100508 (PMC6819742; doi:10.1016/j.ymgmr.2019.100508)
Supplement: Supplementary file 8 — Salford Transition leaflet [file mmc8.pdf]

Salford Royal NHS
NHS Foundation Trust

University Teaching Trust

safe • clean • personal

Transition

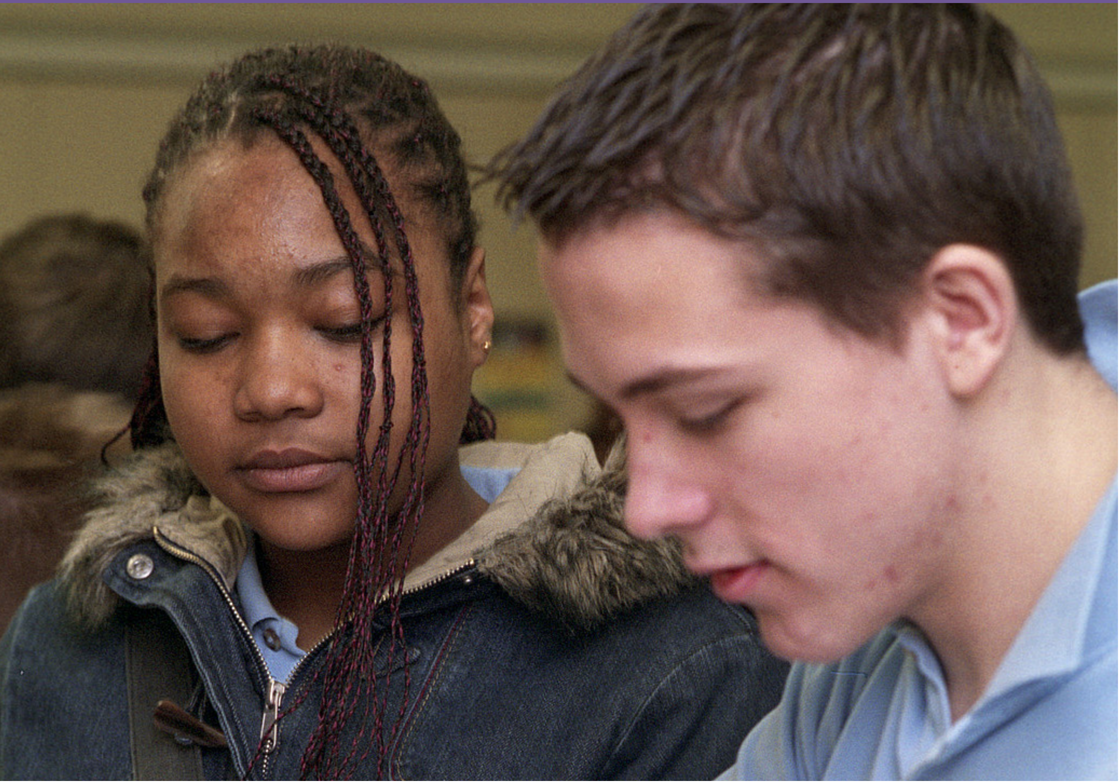

Ladywell Building
Metabolic Unit
0161 206 1899

Health & care information you can trust
The Information Standard Certified Member

© G17040701W. Design Services, Salford Royal NHS Foundation Trust, All Rights Reserved 2017.
Document for issue as handout. Unique Identifier: MED13(17). Review date: April 2019

What is transition?

In health care, the term 'transition' is used to describe the process of planning, preparing and moving from children's health care to adult health care.

Transition is a gradual process of change, which gives time to ensure that you and your family are prepared and feel ready to make the move to adult health care.

Often this occurs when you will also be experiencing other transitions at a similar time, such as moving from school to college or starting work.

It is the opportunity where possible, for you to take responsibility for your own life including healthcare choices.

We recognise that you along with your family will be going through changes in many aspects of your life and that all these changes bring in new people, new styles of communication and new working relationships.

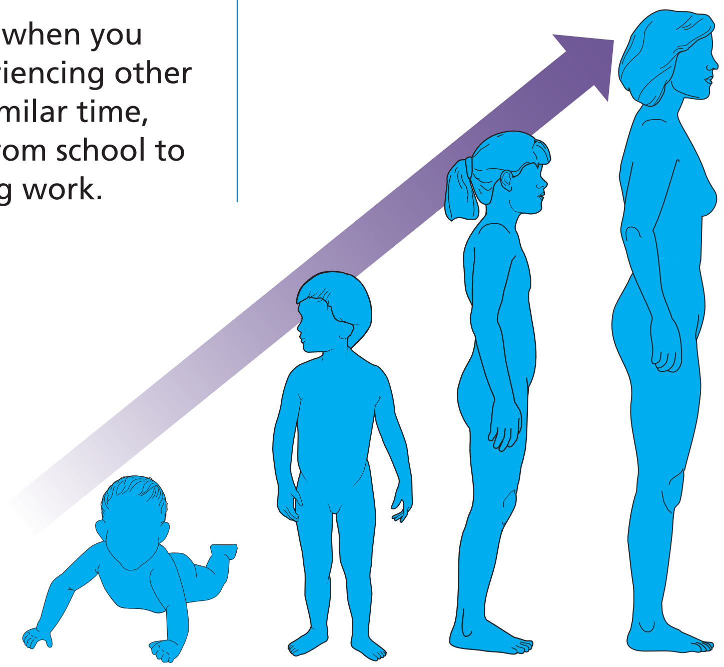

Why do we need to transfer?

As a child you require the skills and knowledge of a paediatrician whose job it is to provide care for babies and children, once you reach adolescence and then adulthood your needs start to change and you require the skills and knowledge of a doctor who is trained to care for adults.

Transition is a natural part of growing up. Transition will be taking place in various areas of your life such as education, social care, financial and other areas of health. Also, legally once you turn 16 you are now able to make decision by yourself regarding your care without your parents.

For those who have capacity issues, this process works differently and our team will work closely with those individuals to ensure that they are supported to be involved in their care as much as possible.

The Mental Capacity Act 2005 will be applied to support these patients appropriately.

Transition is a process and changes will take place at different times for everybody. Families and friends can provide support to young people in gradually developing independence and becoming more involved in their health care. It may be worthwhile starting to consider some of the areas that you may need to start taking more control over:

- Learning the names of regular medicines, why you need them, how much to take and how often
- Asking and answering questions about your health and treatments
- Seeing the doctor or nurse on their own, for part of clinic appointments or consultations
- Staying overnight in hospital without the parent sometimes

© G17040701W. Design Services, Salford Royal NHS Foundation Trust, All Rights Reserved 2017. Document for issue as handout.
Unique Identifier: MED13(17). Review date: April 2019

How does the transition process work?

The transition process can start at any age and this will be decided by your paediatric consultant, although we would usually expect you to be placed in the transition clinic anytime from the age of 14, as this will ensure that transition is a process rather than an immediate change.

We regularly have joint transition clinics, which involves the adult metabolic team from Salford Royal coming across to your children's hospital and carrying out a joint consultation with the children's metabolic team.

This gives the young person the opportunity to meet the adult team in an environment that they are comfortable in and build up a relationship from an early stage; this way when the young person does transfer to the adult service they will be familiar with some members of the team.

It also enables the young person to ask questions that they may have about transition or the adult services. This way the young person is prepared as much as they can be for the change which is about to happen.

As a result of this clinic, a transition plan is agreed between the paediatric consultant and the patient, so that the transfer takes place at a time that is suited to both.

Welcome to the Adult Service!

The adult metabolic service is based at:

Salford Royal NHS Foundation Trust,
Stott Lane,
Salford, M6 8HD

We have out-patient clinics in 3 different areas:

Main out-patients area 6,
Ladywell *and*
Diabetes centre

Our service is a:
Monday-Friday, 9am-5pm service
our clinics run during these hours but we do offer a Monday evening clinic, which some patients prefer to attend due to college/work commitments.

Our team is made up of various professionals including consultant doctors, specialist nurse, nurses, dietitians, research team, pharmacist, physiotherapists, health care assistant, and administrative staff.

The main change is that the focus of the conversation shifts from between the doctor and the parent to between the doctor and you, the patient.

Of course your families' can be involved as much as **YOU** want them to be. Our aim is to make you as independent as possible and we will provide you with the support that you need to achieve this.

This is a long process and will not just happen overnight, everyone is different and will feel ready to be independent at different times.

As an adult service, our purpose is to provide you with the information you require and give you the options so that you can make an informed choice about your healthcare.

© G17040701W. Design Services, Salford Royal NHS Foundation Trust, All Rights Reserved 2017. Document for issue as handout.
Unique Identifier: MED13(17). Review date: April 2019

© G17040701W. Design Services
Salford Royal NHS Foundation Trust
All Rights Reserved 2017
This document **MUST NOT** be photocopied

Information Leaflet Control Policy:
Unique Identifier: MED13(17)
Review Date: April 2019

Salford Royal **NHS**
NHS Foundation Trust

University Teaching Trust

safe • clean • personal

For further information on this leaflet, it's references and sources used, please contact **0161 206 0228**

Copies of this information are available in other languages and formats upon request.

In accordance with the Equality Act we will make 'reasonable adjustments' to enable individuals with disabilities, to access this treatment / service.

If you need this interpreting please telephone

Polish
Jeżeli potrzebne jest Państwu to tłumaczenie, proszę zadzwonić pod numer.

Urdu
اگرآپ کواس ترجمانی کی ضرورت ہے تو مہربانی کرکے فون کریں۔

Arabic
اذا كنتم بحاجة الى تفسير او ترجمة هذا الرجاء الاتصال

Chinese
如果需要翻译，请拨打电话

Farsi
اگر به ترجمه این نیاز دارید ، لطفا تلفن کنید

0161 206 0224

Email: InterpretationandTrans@srft.nhs.uk

Salford Royal operates a smoke-free policy.

For advice on stopping smoking contact the Hospital Specialist Stop Smoking Service on 0161 206 1779

Salford Royal NHS Foundation Trust
Stott Lane, Salford,
Manchester,
M6 8HD
Telephone 0161 789 7373
www.srft.nhs.uk

If you would like to become a Foundation Trust Member please visit:
www.srft.nhs.uk/for-members

If you have any suggestions as to how this document could be improved in the future then please visit:
http://www.srft.nhs.uk/for-patients
