# Supplementary material for: Transition of patients with mucopolysaccharidosis from paediatric to adult care
Source: Mol Genet Metab Rep. 2019 Oct 21;21:100508. doi: 10.1016/j.ymgmr.2019.100508 (PMC6819742; doi:10.1016/j.ymgmr.2019.100508)
Supplement: Supplementary file 9 — Salford Transition plan [file mmc9.pdf]

Transition plan

DATE	SECTION NUMBER	PROGRESS NOTES/GOALS

Transition plan

NOTES

Copyright @ Southampton Children's Hospital, University Hospitals Southampton NHS Foundation Trust 2014. All rights reserved. Users are permitted to use Ready Steady Go and Hello to Adult Services materials in their original format purely for non-commercial purposes. No modifications or changes of any kind are allowed without permission of the copyright holder. The following acknowledgement statement must be included in all publications which make reference to the use of these materials: "Ready Steady Go and Hello to Adult Services developed by the Transition Steering Group led by Dr Arvind Nagra, paediatric nephrologist and clinical lead for transitional care at Southampton Children's Hospital, University Hospitals Southampton NHS Foundation Trust."

Ready Steady Go is based on the work of: 1. S Whitehouse and MC Paone. Contemporary Paediatrics; 1998, 13-16. 2. Paone MC, Wigle M, Saewyc E. Prog Transplant 2006; 16:291-302. 3. Janet E McDonagh et al, J Child Health 2006; 10(1):22-43. Further information at www.uhs.nhs.uk/readysteadygo

NHS

Transition plan

Ready Steady Go programme

Name: ________________ Hospital no: ________________

Address: ________________ DOB: ________________

Transition start date: ________________

School/college: ________________

Home tel no: ________________

Email: ________________ Mobile no: ________________

Diagnosis: ________________

Target date for transfer adult services: ________________

Discharge summary completed: ________________

Resources: ________________ Date: ________________

Transition leaflet for young people ________________

Transition leaflet for parents/carers ________________

Other (please specify) ________________

Offer copy of clinic letters ________________ Date: ________________

MDT involvement

Youth worker ________________

Social worker ________________

Psychology ________________

Specialist nurse ________________

Dietitian ________________

Other

Key worker ________________

Adult unit team contacts ________________

Date of planned visit to adult unit ________________

Transition plan

		R	S	G
**(K)**	**KNOWLEDGE**			
1.	Describes condition, effects and prognosis	☐	☐	☐
2.	Understands medication purpose and effects	☐	☐	☐
3.	Understands treatment purposes and effects	☐	☐	☐
4.	Knows key team members and their roles	☐	☐	☐
**(S)**	**SELF ADVOCACY**			
1.	Part/whole clinic appointment on their own	☐	☐	☐
2.	Knows how to make appointments/alter appointments	☐	☐	☐
3.	Understanding of confidentiality	☐	☐	☐
4.	Orders repeat prescriptions	☐	☐	☐
5.	Takes some/complete responsibility for medication/other treatment	☐	☐	☐
6.	Knows where to get help	☐	☐	☐
**(H)**	**HEALTH AND LIFESTYLE**			
1.	Understands importance of diet/exercise/dental care	☐	☐	☐
2.	Understands impact of smoking/alcohol/substance use	☐	☐	☐
3.	Understands sexual health issues/pregnancy/STDs	☐	☐	☐
**(A)**	**ACTIVITIES OF DAILY LIVING**			
1.	Self care/meal preparation	☐	☐	☐
2.	Independent travel/mobility	☐	☐	☐
3.	Trips/overnight stays away from home	☐	☐	☐
4.	Benefits	☐	☐	☐

Transition plan

		R	S	G
**(V)**	**VOCATIONAL**			
1.	Current and future education/impact of condition on career plans	☐	☐	☐
2.	School attendance and performance	☐	☐	☐
3.	Work experience and how to access careers advice	☐	☐	☐
4.	Outside activities and interests	☐	☐	☐
5.	Disclosure to school/employer	☐	☐	☐
**(P)**	**PSYCHOSOCIAL**			
1.	Self esteem/self confidence	☐	☐	☐
2.	Body/self image	☐	☐	☐
3.	Peer relationships/bullying	☐	☐	☐
4.	Support networks/family/disclosure to friends	☐	☐	☐
5.	Coping strategies	☐	☐	☐
**(T)**	**TRANSITION**			
1.	Understands concept of transition	☐	☐	☐
2.	Agrees transition plan	☐	☐	☐
3.	Attends transition clinic	☐	☐	☐
4.	Visits adult unit (if appropriate)	☐	☐	☐
5.	Sees GP independently	☐	☐	☐

Ready Date ____________ Signature ____________

Steady Date ____________ Signature ____________

Go Date ____________ Signature ____________

Transition plan

Please tick and date when addressed, details to be recorded in free text section giving code eg K2, A3

DATE	SECTION NUMBER	PROGRESS NOTES/GOALS
